# Commentary: The ME-BYO index: A development and validation project of a novel comprehensive health index

**DOI:** 10.3389/fpubh.2024.1495056

**Published:** 2025-01-07

**Authors:** Nobunao Ikewaki, Koji Ichiyama, Rajappa Senthilkumar, Senthilkumar Preethy, Samuel J. K. Abraham

**Affiliations:** ^1^Department of Medical Life Science, Kyushu University of Medical Sciences, Nobeoka, Miyazaki, Japan; ^2^Institute of Immunology, Junsei Educational Institute, Nobeoka, Miyazaki, Japan; ^3^Antony- Xavier Interdisciplinary Scholastics (AXIS), GN Corporation Co. Ltd., Kofu, Japan; ^4^Fujio-Eiji Academic Terrain (FEAT), Nichi-In Centre for Regenerative Medicine (NCRM), Chennai, India; ^5^Centre for Advancing Clinical Research (CACR), University of Yamanashi-School of Medicine, Chuo, Japan; ^6^Mary-Yoshio Translational Hexagon (MYTH), Nichi-In Centre for Regenerative Medicine (NCRM), Chennai, India; ^7^R & D, Sophy Inc., Nyodogawa, Japan; ^8^Levy-Jurgen Transdisciplinary Exploratory (LJTE), Global Niche Corp., Wilmington, DE, United States; ^9^Haraguchi-Parikumar Advanced Remedies (HARP), SoulSynergy Ltd., Vacoas-Phoenix, Mauritius

**Keywords:** neutrophil-to-lymphocyte ratio (NLR), ME-BYO, immunity, NLR modifying glucan, health index, beta 1,3-1,6 glucan, beta-glucan

The article by Nakamura et al. on the ME-BYO index (1) presents a ground breaking approach rooted in the “Healthcare New Frontier” policy initiative of Kanagawa Prefecture in Japan. This innovative concept introduces the ME-BYO index as a tool to measure and visualize the dynamic changes in an individual's health status. ME-BYO, translating roughly to “no disease,” represents the transitional phase between health and the onset of disease or disability. It emphasizes the importance of early intervention, as preventive strategies during this transition can effectively maintain or even reverse disease progression (1).

The ME-BYO index is quantified using a 15-item measure that evaluates four key domains: metabolic function, locomotor function, cognitive function, and mental resilience. This comprehensive assessment offers a unique perspective on monitoring health in real time (1). However, we wish to highlight two critical aspects that can further refine the clinical application of the ME-BYO index:

1. Neutrophil-to-lymphocyte ratio (NLR) as a vital disease indicator

The NLR is an accessible and reliable marker of the immune response to both infectious and non-infectious stimuli. It provides a snapshot of the balance between innate immunity (represented by neutrophils) and adaptive cellular immunity (represented by lymphocytes). NLR is influenced by numerous factors, including age, race, medications, and chronic conditions such as coronary heart disease, stroke, diabetes, obesity, psychiatric disorders, solid organ cancers, anemia, and stress (2).

The normal range of NLR in healthy adults typically falls between 1 and 2, while values above 3.0 or below 0.7 are often pathological. Importantly, an NLR in the “gray zone” of 2.3–3.0 may serve as an early warning signal for various pathological processes such as cancer, atherosclerosis, infection, inflammation, psychiatric disorders, and stress. NLR has been well-established as a prognostic marker, independently correlating with mortality in both the general population and specific disease groups (e.g., sepsis, pneumonia, COVID-19, cancer). Moreover, NLR has recently gained traction in clinical decision-making, particularly in managing patients with COVID-19 pneumonia (3).

2. A safe intervention in beneficially modifying NLR

Building on the ME-BYO index's objective to encourage individuals to adopt preventive and therapeutic measures during the transition to disease states, we propose the inclusion of beta-glucans as biological response modifiers (BRMGs). Our research over the past decade has focused on investigating the health promoting potentials of these BRMGs. Produced by two novel strains of *Aureobasidium pullulans* (AFO-202 and N-163), these beta-glucans are safe, allergen-free, and produced under GMP conditions in Japan. As nutritional supplements, beta-glucans have demonstrated potential in enhancing immune function and improving various health outcomes. The AFO-202 BRMG, has shown significant effects in metabolic regulation and immune enhancement across a range of conditions. Pre-clinical and clinical studies have demonstrated its efficacy in metabolic disorders such as diabetes, dyslipidemia, and non-alcoholic steatohepatitis (NASH), as well as in neurodevelopmental disorders like autism and neurodegenerative diseases such as Parkinson's disease (4–9). Additionally, AFO-202 in combination with N-163 has shown promising results in infectious diseases, including COVID-19 (10, 11). On the other hand, the N-163 BRMG has exhibited potential as an immune modulator in NASH, COVID-19, muscle-wasting diseases such as Duchenne muscular dystrophy, and autoimmune conditions like multiple sclerosis and psoriasis (9–15). Both BRMGs, alone or in combination, have demonstrated the ability to modulate the gut microbiome and its metabolites, impacting various disease states while promoting overall health (12, 16, 17). Notably, they have shown efficacy in regulating the neutrophil-to-lymphocyte ratio (NLR) in conditions such as metabolic disorders, cancer, and psoriasis (15, 18, 19). In these studies, the modulation of NLR was particularly pronounced in individuals with elevated baseline NLR levels, underscoring the potential of beta-glucans to regulate immune function even in advanced disease states (20).

The ability of these beta-glucans to influence NLR, especially in diseases like cancer and psoriasis (18–20), highlights their potential as a therapeutic tool for immune modulation. The observed reduction in elevated NLR levels in these conditions further reinforces their role in balancing immune responses, potentially aiding in the transition back to health as envisioned in the ME-BYO framework. Given the close relationship between NLR and immune function, beta-glucans could potentially modulate NLR and thereby influence the ME-BYO index during critical transitions in health. This approach aligns with the ME-BYO index's focus on early intervention, encouraging individuals to take proactive steps to maintain health and prevent disease progression.

In conclusion, the ME-BYO index is a powerful tool for visualizing and managing health transitions. By incorporating NLR as a key indicator and exploring the potential of beta-glucans as immune modulators, the ME-BYO concept can further be positioned as an yardstick to measure the outcome of several other interventions, while safe modifying interventions such as these beta glucans could be proposed as universal NLR modifiers which demonstrate potential for preventing several pre-disease conditions from evolving into full blown disease.
